# The Impact of Temperature and the Duration of Freezing on a Hydrogel Used for a 3D-Bioprinted In Vitro Skin Model

**DOI:** 10.3390/biomedicines12092028

**Published:** 2024-09-05

**Authors:** Maja Sever, Dominik Škrinjar, Tina Maver, Monika Belak, Franc Zupanič, Ivan Anžel, Tanja Zidarič

**Affiliations:** 1 Institute of Biomedical Sciences, Faculty of Medicine, University of Maribor, Taborska Ulica 8, 2000 Maribor, Slovenia; maja.sever@student.um.si (M.S.); dominik.skrinjar@student.um.si (D.Š.); monika.belak@um.si (M.B.); tanja.zidaric@um.si (T.Z.); 2 Department of Pharmacology, Faculty of Medicine, University of Maribor, Taborska Ulica 8, 2000 Maribor, Slovenia; 3 Faculty of Mechanical Engineering, University of Maribor, Smetanova 17, 2000 Maribor, Slovenia; franc.zupanic@um.si (F.Z.); ivan.anzel@um.si (I.A.)

**Keywords:** in vitro skin model, 3D printing, hydrogels, preclinical and clinical medicine

## Abstract

Skin bioprinting has the potential to revolutionize treatment approaches for injuries and surgical procedures, while also providing a valuable platform for assessing and screening cosmetic and pharmaceutical products. This technology offers key advantages, including flexibility and reproducibility, which enable the creation of complex, multilayered scaffolds that closely mimic the intricate microenvironment of native skin tissue. The development of an ideal hydrogel is critical for the successful bioprinting of these scaffolds with incorporated cells. In this study, we used a hydrogel formulation developed in our laboratory to fabricate a 3D-bioprinted skin model. The hydrogel composition was carefully selected based on its high compatibility with human skin cells, incorporating alginate, methyl cellulose, and nanofibrillated cellulose. One of the critical challenges in this process, particularly for its commercialization and large-scale production, is ensuring consistency with minimal batch-to-batch variations. To address this, we explored methods with which to preserve the physicochemical properties of the hydrogels, with a focus on freezing techniques. We validated the pre-frozen hydrogels’ printability, rheology, and mechanical and surface properties. Our results revealed that extended freezing times significantly reduced the viscosity of the formulations due to ice crystal formation, leading to a redistribution of the polymer chains. This reduction in viscosity resulted in a more challenging extrusion and increased macro- and microporosity of the hydrogels, as confirmed by nanoCT imaging. The increased porosity led to greater water uptake, swelling, compromised scaffold integrity, and altered degradation kinetics. The insights gained from this study lay a solid foundation for advancing the development of an in vitro skin model with promising applications in preclinical and clinical research.

## 1. Introduction

As the body’s largest organ, the skin is a crucial barrier that protects the internal organs from various external threats. It also provides essential sensory feedback, enabling interaction with the environment. The complex structure of the skin facilitates critical cell–cell interactions, including paracrine and autocrine signaling, and plays a vital role in regulating body temperature and homeostasis [1,2,3,4,5].

A disruption of the skin barrier can lead to severe physiological consequences, such as fluid loss, inflammation, and a heightened risk of infection. Open wounds are prone to bacterial colonization, which can result in sepsis and life-threatening conditions. The healing process often leads to the formation of scar tissue, which lacks the functional properties of intact skin [2,4,5,6].

Despite advances in wound healing, autologous skin grafts remain the gold standard for treating extensive wounds. However, this approach has limitations, including a shortage of donor skin and potential complications, such as scarring, infection, immune rejection, and bleeding. These challenges underscore the need for alternative solutions for skin regeneration and wound care [4,5,7,8].

In response, three-dimensional (3D) in vitro skin models are gaining prominence in regenerative medicine. These models are invaluable in wound healing and fundamental preclinical research, particularly in the pharmaceutical and cosmetic industries. They are becoming increasingly important tools for improving our understanding of the physiology and functioning of the skin. In addition, they play a crucial role in certain applications, such as the toxicity testing of dermatological substances, the evaluation of non-invasive sensors and medical devices, and the assessment of (trans)dermal drugs and cosmetic products [9]. By using human cells, these models more accurately mimic human skin physiology, reducing the reliance on animal testing and addressing ethical concerns [1,2,3,5,10].

Three-dimensional bioprinting technology has revolutionized tissue engineering by enabling the precise layer-by-layer replication of biological and mechanical properties. This technology allows for incorporating bioactive substances and cells into polymers, facilitating the creation of intricate scaffold structures. These advancements have accelerated healing and have paved the way for personalized wound dressings and skin tissue engineering solutions [1,4,5].

Hydrogels, often derived from biocompatible polymers like alginate (ALG) and methylcellulose (MC), are particularly appealing for cell encapsulation in 3D bioprinting. However, their use poses a challenge, mainly due to their suboptimal printability. These hydrogels often have a low viscosity and have difficulty retaining their shape after deposition, which limits their effectiveness. In addition, the limited choice of hydrogels hinders the further development of 3D printing with hydrogels for tissue engineering applications [11]. Enhancing the viscosity of the precursor solutions with modifiers, or combining hydrogels with other materials, such as cellulose nanofibrils, could improve printability and the overall properties of the final construct [5,12,13,14].

Hydrogels possess three-dimensional network structures, providing natural softness, high flexibility, elasticity, biodegradability, self-healing properties, and the capacity to absorb significant amounts of water. These attributes make them ideal for drug delivery systems, regenerative medicine, water purification, and mechanical and biofunctional engineering. Additionally, hydrogels are highly responsive to external factors, such as pH, temperature, and mechanical forces, making them particularly valuable in the field of flexible electronics. Recently, there has been a rapid surge in the development of hydrogel-based applications for smart devices, soft robotics, healthcare, and human–machine interfaces [15].

In vitro 3D human skin models are essential for deepening our skin physiology and function knowledge. However, many of the current reconstructed models lack the structural complexity needed to accurately mimic native human skin. To overcome these limitations, hydrogels have emerged as effective scaffold materials for creating dermal equivalent 3D skin models, offering enhanced flexibility, better control over the scaffold properties, and improved integration of the cellular components [9].

Creating in vitro skin models that closely replicate native human skin offers a promising alternative to animal models and enables more precise and clinically relevant testing. Advances in biofabrication techniques and biomaterials have led to the development of increasingly sophisticated, multilayered skin models that include key functional components, such as the skin barrier, mechanical properties, pigmentation, blood vessels, hair follicles, glands, and subcutaneous layer. This enhanced capacity to mimic the functional characteristics of native skin improves the modeling of native human skin behavior and responses by incorporating the complex interactions between cells and their surrounding materials [16].

A significant challenge to the development of consistent 3D-bioprinted skin models is the variability in hydrogel batches, which can impact the reproducibility of the models’ structural integrity and functional performance. To address this issue, our study aimed to determine whether the large-scale production and subsequent freezing of hydrogel could preserve its critical printability and other essential properties. Hydrogels are particularly vulnerable to low temperatures, given their high water content, which may compromise their functionality when stored at sub-freezing conditions. Although some studies have included the properties of hydrogels at low temperatures, these have primarily focused on developing anti-freezing hydrogels through various synthesis strategies [17,18,19,20]. To the best of our knowledge, no studies have specifically examined the feasibility of 3D printing of pre-frozen hydrogels for fabricating any in vitro tissue model.

This research investigates the effects of freezing on the physicochemical properties of hydrogels, including their viscosity, porosity, mechanical properties, swelling, and degradation kinetics. By examining the impact of temperature and storage duration—specifically, freezing at −20 °C and −80 °C for 7 and 29 days—we sought to determine whether the key hydrogel properties remained consistent post-thaw compared to freshly prepared hydrogels. Confirming this hypothesis would enable the use of a single hydrogel batch across all the stages of in vitro skin model development, thereby eliminating the potential variations that can arise from a manual preparation process, such as differences in mixing or room temperature.

The findings from this study are crucial for advancing the reproducibility and precision of 3D-bioprinted in vitro skin models. By ensuring the consistency of the hydrogel material used, this research lays the groundwork for more reliable and scalable applications in regenerative medicine and related fields, ultimately contributing to the development of robust in vitro models for preclinical and clinical research.

## 2. Materials and Methods

### 2.1. Materials

The sodium salt of alginic acid (ALG; Mw: 80 kDa), methylcellulose (MC; viscosity: 1500 cP), and calcium chloride (CaCl_2_, (>93% purity)) for the hydrogel and subsequent scaffold production were purchased from Sigma-Aldrich (Taufkirchen, Germany). Additional materials obtained from Sigma-Aldrich, Taufkirchen, Germany, included L-glutamine (>99% purity), penicillin G sodium salt, and streptomycin sulfate salt (~95% purity). A suspension of cellulose nanofibrils (NFC, 3% (*w*/*v*) suspensions in water) was purchased from The Process Development Center, University of Maine (Orono, ME, USA). Advanced Dulbecco’s Modified Eagle Medium (ADMEM) and fetal bovine serum (FBS, premium grade) were acquired from Thermo Fisher Scientific (Schwerte, Germany). All the materials were used as received without further modification. Ultra-pure water (18.2 mΩ cm at 25 °C), produced by an ELGA Purelab water purification system (Veolia Water Technologies, High Wycombe, UK), was used to prepare the CaCl_2_ solutions.

### 2.2. Preparation of the Hydrogel for 3D Printing

The hydrogel formulation used in this study was previously tested and optimized for developing in vitro skin models and customizable dressings [5,13,21]. As our focus was on investigating the effects of different storage conditions on the physicochemical properties of the polymer base material, human skin fibroblasts were not incorporated in situ. The optimized hydrogel formulation, consisting of 3 wt% ALG, 6 wt% MC, and 1.5 wt% NFC, was printable and maintained the integrity of the 3D-printed geometry. For this study, a portion of the prepared ALG-MC-NFC (AMN) hydrogel base was stored at −20 °C and −80 °C, while freshly prepared AMN hydrogel was used as the control (CTRL).

### 2.3. Three-Dimensional Bioprinting and Postprocessing

#### 2.3.1. Three-Dimensional Bioprinting

**Figure 1:**
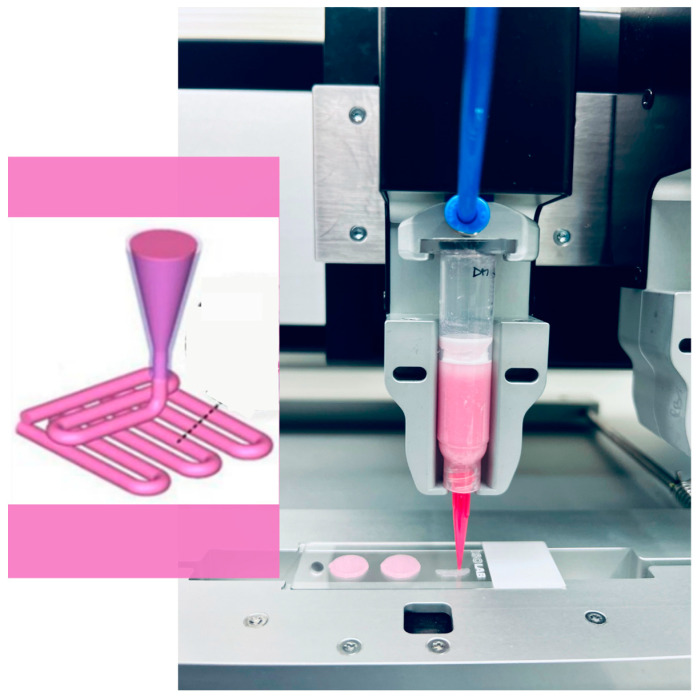
Schematic presentation of 3D bioprinting with layer-by-layer approach (**left**) and fabrication of AMN scaffolds (**right**). Reprinted from ref. [22].

We fabricated 4-layer scaffolds with a 3D architecture using an extrusion-based bioprinting process. The scaffolds were created using a VitaPrint 3D bioprinter (IRNAS Institute, Maribor, Slovenia) capable of producing pores with precise sizes, shapes, and densities in the z-direction. We used extrusion nozzles (Nordon EFD, East Providence, RI, USA) with a diameter of 0.25 mm. Cylinder-shaped scaffolds (10 mm in diameter, 0.8 mm in height) were initially modeled using a Scaffold Generator v5 (Institute IRNAS, Maribor, Slovenia). The scaffolds were fabricated layer by layer, with the layers deposited in a 0°/90° pattern on a substrate (Figure 1). The scaffolds that failed to maintain their structural integrity and geometry were excluded from further analysis.

After printing, all the scaffolds were cross-linked using a 1% CaCl_2_ solution. The scaffolds were immersed in the CaCl_2_ solution for 5 min, which was sufficient to facilitate the formation of ionic bonds between the ALG and NFC. As polysaccharides with negatively charged carboxylic groups on their surface, ALG and NFC were chosen for their intrinsic ability, as carboxylated polymers, to form electrostatic and hydrogen bonds with bivalent calcium ions. This cross-linking step ensured that the 3D-printed scaffolds retained their mechanical stability due to their fibrillar structure [5,23,24,25].

#### 2.3.2. Lyophilization

Before lyophilization, the cross-linked samples were frozen overnight at −80 °C. The frozen samples were then transferred to a pre-cooled lyophilizer (VirTis 6K BenchTop, Scientific products, Warminster, PA, USA) and lyophilized overnight at a final temperature of −104 °C and pressure of 98 μBar. After the lyophilization process, the lyophilized scaffolds were hermetically sealed until testing.

### 2.4. Scaffold Characterization Methods

#### 2.4.1. Rheological Measurements

The rheological properties of the hydrogel base materials were evaluated using a Rheolab QC rheometer (Anton Paar, Graz, Austria) equipped with a CC27-SN25789 cylinder measurement system (Anton Paar, Graz, Austria), following the ISO 3219 standard [26,27]. The measurements were conducted at a constant temperature of 25 °C. The shear rate (Γ) was progressively increased from 0.01 to 1000 s^−1^, with 31 measuring points recorded every 10 s over 310 s. The Carreau–Yasuda model was employed for data analysis and curve fitting [28].

#### 2.4.2. Attenuated Total Reflectance Infrared Spectroscopy (AFT-IR)

The chemical composition of all the lyophilized samples was analyzed using attenuated total reflectance infrared spectroscopy (ATR-IR) with an Agilent Cary 630 FTIR spectrometer (Agilent, Santa Clara, CA, USA). The measurements were conducted at room temperature using a diamond ATR module. A small amount of the lyophilized, 3D-printed samples, sufficient to cover the ATR crystal surface, was placed on the crystal for analysis. The IR spectra for the CTRL and pre-frozen samples were recorded over a 4000–650 cm^−1^ range with a resolution of 4 cm^−1^. Each spectrum was generated from 32 scans, with eight replicate measurements for each sample [13,29,30]. The IR data were processed and analyzed using MicroLab PC 4.0 software (Agilent, Santa Clara, CA, USA), and the final absorbance versus the wavenumber plots were generated using OriginPro 8.5 (OriginLab, Stoke Mandeville, Buckinghamshire, UK).

#### 2.4.3. Swelling Test

The swelling kinetics of the scaffolds were investigated using the gravimetric method [31]. The dried scaffolds were initially weighed (*w*_0_) and then immersed in ADMEM supplemented with 5% FBS at 37 °C. At predetermined time intervals, the samples were removed from the medium, gently blotted with filter paper to remove excess surface liquid, and weighed (*w_t_*). The swelling ratio at each time point was calculated using Equation (1):

(1) $$Swelling\;ratio=\frac{w_{t}{-w}_{0}}{w_{0}}\times 100\%$$

where $w_{0}$ and $w_{t}$ represent the initial dry weight and the weight of the swollen scaffolds at time *t*, respectively. The measurements were performed in triplicate, and the mean values were calculated and reported along with the standard error.

#### 2.4.4. In Vitro Degradation Test

Before the weight loss test, the cylinder-shaped scaffolds were lyophilized and initially weighed (*w*_0_). They were then immersed in ADMEM supplemented with 5% FBS at 37 °C. The scaffolds were removed from the medium at predetermined intervals, lyophilized again, and weighed (*w_t_*). The remaining weight of the scaffolds was calculated using the following Equation (2) [31,32]:

(2) $$Weight\;remaining=\frac{w_{t}}{w_{0}}\times 100\%$$

where $w_{0}$ represents the initial weight and $w_{t}$ is the remaining weight of the scaffold at time *t*. The measurements were performed in triplicate, and the results are presented as the mean values with standard error and plotted accordingly.

#### 2.4.5. Internal Structure Analysis Using Nano-Computed Tomography

Nano-computed tomography (NanoCT) was employed to evaluate the impact of freezing on the microstructure and porosity of the materials. Imaging was performed using a ZEISS Xradia 620 Versa 3D X-ray microscope (Carl Zeiss AG, Oberkochen, Germany). Before the analysis, the 3D-printed scaffolds were frozen at −80 °C and freeze-dried. Measurements were taken using a 4× objective at 60 kV and 6.50 W, with an LE #1 filter applied. The exposure time was set to 1.0 s, with the binning configured to 2, resulting in a voxel size of 2.70 μm. Image reconstruction was performed using ZEISS Xradia TXM3DViewer v1.2.10 software (Carl Zeiss AG, Oberkochen, Germany), and further analysis was conducted with NIH ImageJ 1.52g software (NIH, Bethesda, MD, USA) [21].

#### 2.4.6. Morphology and Internal Structure Analysis Using Scanning Electron Microscopy

The morphology of the samples was examined using Field Emission Scanning Electron Microscopy (FESEM, Carl Zeiss FE-SEM SUPRA 35 VP electron microscope, Zeiss, Germany). Before the imaging of the aluminum measurements, all the samples were lyophilized, mounted individually, and placed on aluminum SEM sample holders using double-sided conductive carbon tape. The SEM images were acquired at an accelerating voltage of 1 keV and a working distance of approximately 4.5.

#### 2.4.7. Mechanical Properties Analysis

Indentation tests were conducted using a Nano Test Vantage (Micro Materials Limited, Wrexham, UK). The indentation load was generated by an electromagnetic force (resolution 3 nN), and the indentation displacement was measured by the change in capacitance (resolution 0.002 nm). A Berkovich indenter was used to measure the indentation properties as follows: indentation hardness, indentation elastic modulus, plastic, and elastic work. The loading time, up to a maximum load of 20 mN, was 10 s; the time at maximum load was 3 s; and the unloading time was 1 s. For each sample, 25 measurements were taken, with a sample spacing of 50 µm.

#### 2.4.8. Statistical Analysis

The numerical values are presented as the mean ± standard deviation (SD). A Shapiro–Wilk test confirmed that the experimental data followed a normal distribution. Levene’s test was then applied to verify the equality of variances. Since all the data sets exhibited a normal distribution and homoscedasticity, a one-way analysis of variance (ANOVA) followed by a Bonferroni post hoc test were conducted. P-values less than 0.05 were considered statistically significant. Statistical analyses were performed using SPSS Statistics 27 (IBM Corp., Armonk, NY, USA).

## 3. Results and Discussion

The three-dimensional bioprinting of in vitro skin models represents a promising preclinical and clinical studies technology. These engineered skin models offer new ways to closely mimic native skin, providing a more ethical alternative to animal testing [33]. Extrusion bioprinting technology, which leverages various materials, has been effectively used to replicate the complexity of human skin. Hydrogels, composed of cross-linked solid and liquid phases, are particularly well suited for constructing soft tissues, due to their similarity to the soft tissues found in the human body. However, their high water content produces a low proportion of the load-bearing solid phase, leading to their weak and fragile mechanical properties [34].

The physical properties and stimuli of the microenvironment significantly influence cell behavior. Therefore, when developing materials with which to mimic the ECM of tissues, the careful consideration of their mechanical properties is essential [35]. Additionally, in research it is crucial that the methods are reproducible and repeatable, and that the data are derived from samples that are as comparable as possible [36]. A critical factor for the broader adoption of 3D-printed in vitro skin models is the ability to mass produce them consistently. To ensure this, we produced large quantities of the base material, which was not used immediately. The base material from the same batch must have consistent physicochemical and mechanical properties, regardless of the storage conditions (e.g., freezing), to ensure the reproducibility of the 3D-printed in vitro models. With this in mind, we investigated whether freezing alters the arrangement of the polymer chains in the hydrogel, and consequently affects its physicochemical (swelling, degradation) and biological properties.

### 3.1. Printability of the Prepared Hydrogel Formulation

The rheological properties of hydrogels play a crucial role in determining printability, which refers to the ability to extrude the desired 3D structures while maintaining their geometry [37]. The composition of the hydrogel is a significant factor that influences these fundamental rheological properties, affecting the bioprinting process in terms of controlling the resolution and shape fidelity of the 3D-printed structures [13,38,39].

Most naturally derived hydrogels possess a low shear modulus, indicating their soft and flexible nature. Such materials exhibit characteristics like those of highly viscous fluids (e.g., honey), which can flow or deform slowly under stress. Viscosity plays a critical role in layer-by-layer build-up during printing, and can potentially deform the precise formation of complex 3D structures—generally, a higher viscosity leads to improved printing fidelity. However, an excessively high viscosity may require increased extrusion pressure, which can adversely affect the optimal behavior of the cells within the bioink [39,40].

**Figure 2:**
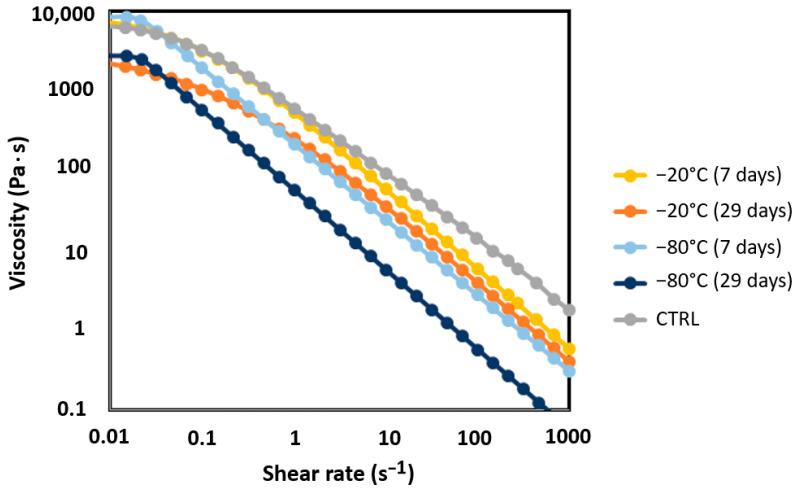
Viscosity measurements of the AMN-based hydrogel. The graph shows the measurements for the control (CTRL) hydrogel, which is the fresh hydrogel, and the hydrogels frozen for 7 or 29 days at −20 °C and −80 °C.

As previously mentioned, the arrangement of the polymer chains in hydrogels can be influenced by the formation of water crystals during freezing, which, in turn, affects the material’s extrusion properties. The rheological properties of the prepared hydrogels were examined to assess the impact that freezing the base hydrogel has on printability (Figure 2). Many researchers often use the rheological characterizations as a proxy for extrudability, with viscosity being the primary measure for this purpose; a higher viscosity generally correlates with lower extrudability. Since most hydrogels exhibit non-Newtonian behavior (i.e., viscosity decreases as shear rate increases), it is essential to measure the viscosity across a wide range of shear rates, particularly at the upper end, where the shear rate approximates the conditions experienced by hydrogels during extrusion [41].

The shear-thinning behavior is closely related to the viscoelastic properties of a material, specifically the storage modulus (G′) and loss modulus (G″), as both reflect the material’s internal structure and its response to deformation. As the shear rate increases, the viscoelastic properties of shear-thinning materials change, transitioning from a more solid-like to a more liquid-like state. When a shear-thinning material is subjected to oscillatory shear, as in rheological measurements, its viscoelastic properties can vary with the shear rate. The material may exhibit a higher storage modulus (G′) at low shear rates due to a more intact internal structure (e.g., polymer network), indicating solid-like behavior. As the shear rate increases, the structure begins to break down or align, reducing the viscosity (shear thinning) and possibly decreasing the G′.

In practical terms, hydrogels with a dominant elastic behavior (high G′) are difficult to extrude through a nozzle because they resist flow. Conversely, if a hydrogel is too viscous (high G″), it may extrude easily but fail to retain its shape after extrusion [42,43,44,45,46].

The combination of the selected polymers, ALG, MC, and NFC, exhibits pseudoplastic behavior, which is characteristic of polymer solutions. As shown in Figure 2, all hydrogels demonstrate shear-thinning behavior, indicated by a decrease in the viscosity as the shear rate increases, which helps to maintain the printed shape [39,47]. This occurs because, at higher shear rates, the shear forces align the macromolecules more strongly in the direction of the flow, reducing flow resistance.

The observed reduction in viscosity can be attributed to the combined effects of the electrostatic and steric repulsion between the charged groups of ALG and NFC within the network under shear forces. Additionally, the anionic carboxyl groups in ALG contribute to the dehydration of MC, resulting in the reorientation of the molecular chain arrangement [13,48,49]. This behavior facilitates the smooth extrusion of the hydrogel through the nozzle during 3D bioprinting, minimizing the risk of clogging while preserving the hydrogel’s original shape, even under significant shear forces [5,39].

**Figure 3:**
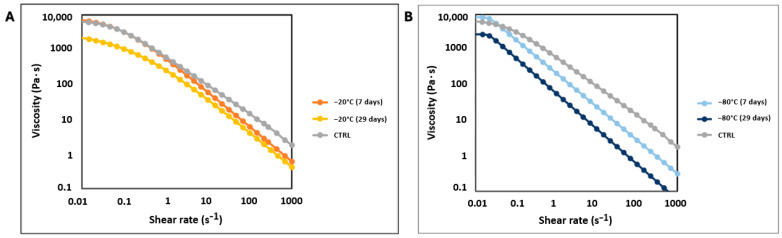
Viscosity measurements of the AMN-based hydrogel. The graph shows the measurements for the CTRL hydrogel, which is the fresh hydrogel, and the hydrogels frozen for (**A**) 7 or 29 days at −20 °C and (**B**) 7 or 29 days at −20 °C.

As shown in Figure 2 (with more evident differences in shape depicted in Figure 3A,B), the flow curves of the AMN samples vary in shape, likely due to the microstructural properties of the AMN hydrogels. Both the freshly prepared CTRL hydrogel and the AMN hydrogels frozen at −20 °C exhibit shear-thinning behavior across the entire shear rate range (Figure 3A). However, the samples frozen at −80 °C (Figure 3B) maintain a constant shear viscosity at low shear rates.

The constant zero-shear viscosity at low shear rates occurs because the rate at which the shear forces separate the molecules is nearly balanced by the rate at which they form new bonds. This zero-shear viscosity reflects the overall structure of the biopolymers, particularly how the side branches of the carbohydrate polymers become entangled. As the shear rate increases, the molecules are pulled apart more quickly than they can re-entangle, causing the macromolecules to align more with the flow direction, thereby reducing flow resistance [13].

The storage conditions of the AMN hydrogels influenced the shape of their flow curves and significantly affected their viscosity. Although all the pre-frozen AMN hydrogels (frozen for 7 or 29 days at −20 °C and −80 °C, respectively) exhibited pseudoplastic behavior similar to that of the CTRL samples (particularly those frozen at −20 °C), their viscosity was notably reduced. This reduction in viscosity was more pronounced for the hydrogels frozen at −80 °C, especially those frozen for 29 days, compared to those frozen for just 7 days.

For instance, the CTRL hydrogel had a viscosity of approximately 100 Pa s at 10 s^−1^, and gradually decreased to around 14 Pa s at 100 s^−1^. In comparison, the hydrogel frozen for 7 days at −20 °C had a viscosity of about 60 Pa s at 10 s^−1^ and 6 Pa s at 100 s^−1^. For the samples frozen at −80 °C, the viscosity values dropped to 24.5 Pa s and 3 Pa s at 100 s^−1^. The influence of prolonged storage is evident from the further viscosity decreases: for the hydrogels frozen for 29 days at −20 °C, the viscosity dropped to 35.9 Pa s at 10 s^−1^ and 4 Pa at 100 s^−1^, while those frozen at −80 °C saw a reduction to 5.82 Pa s at 10 s^−1^ and 0.58 Pa s at 100 s^−1^. These results indicate that both the duration and temperature of freezing significantly reduce hydrogel viscosity, with the most substantial decreases being observed after extended freezing periods at lower temperatures. This reduction in viscosity is associated with the redistribution of polymer chains and the crystallization that occur during the freezing process, leading to physical cross-linking. This process results in the more ordered structure of the polymer chains within the hydrogel, which reduces their ability to align under shear forces and makes them less extrudable. Consequently, the hydrogel exhibits a lower shear modulus [13,50].

The altered rheological properties, including the reduced viscosity due to polymer chain redistribution and crystallization, adversely affect the printability of the hydrogel. The lower viscosity of frozen AMN hydrogels may also impact the gel’s surface charge and branching, due to potential electrostatic repulsion. This could explain the challenges encountered during 3D printing, such as difficulty extruding the hydrogel through the nozzle; frequent nozzle blockages; and structural deficiencies in the printed 3D scaffolds, such as missing layers and increased brittleness; that ultimately undermine the mechanical integrity of the scaffold. Despite these challenges, the frozen hydrogels retain sufficient shear-thinning properties to enable the 3D printing of scaffolds with the desired shape.

### 3.2. Chemical Composition of 3D-Bioprinted Scaffolds

**Figure 4:**
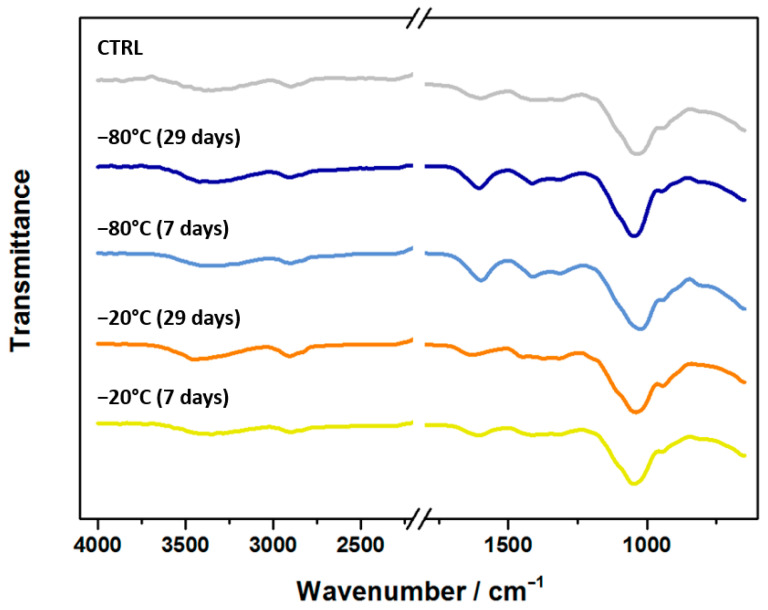
FTIR spectra of 3D-bioprinted scaffolds prepared from fresh hydrogel (CTRL) and hydrogels frozen for 7 or 29 days at −20 °C and −80 °C.

The chemical composition and potential effects of a rearranged polymer network in the 3D-bioprinted scaffolds prepared from pre-frozen hydrogels were analyzed using FTIR (Figure 4).

As expected, the structural features of all the samples, including the position of the characteristic peaks, remained consistent, indicating that the formation of ice crystals did not alter the chemical composition of the pre-frozen samples. The broad band observed between 3500 and 3000 cm^−1^ corresponds to O-H stretching vibrations, likely due to adsorbed water [13,51,52]. The 1400 to 900 cm^−1^ region is the fingerprint region of cellulose derivatives, such as methylcellulose (MC) and nanofibrillated cellulose (NFC). Within this region, the bands in the 1450–1350 cm^−1^ range are attributed to the symmetric bending vibrations of the methylene groups and C-H vibrations in the pyranose ring, while the bands between 1390 and 1300 cm^−1^ are associated with O-H vibrations [13,53,54]. The C-O stretching vibrations of the primary and secondary alcohol groups in cellulose and its derivatives are detected at 1110 and 1050 cm^−1^ [13,53]. Additionally, a band observed in the 2850–2910 cm^−1^ range corresponds to the C-H stretching mode of alkyl groups. The saccharide structure of alginate (ALG) is reflected in the bands around 1300 and 1020 cm^−1^, which correspond to the C-O stretching vibrations and C-O-C vibrations of the mannuronic and guluronic units, respectively [13,55]. Furthermore, alginate shows bands in the range of 1600–1550 cm^−1^ and around 1415 cm^−1^, which are associated with the antisymmetric and symmetric stretching vibrations of the carboxylate groups in its backbone [13,55,56].

### 3.3. Three-Dimensional-Bioprinted Scaffolds Water Uptake Capacity (Swelling Ratio)

An effective in vitro skin model should closely mimic natural skin, making it crucial to understand the properties of the hydrogel scaffold, their dynamic interactions, and how these factors influence the structure and function of the mature tissue-engineered skin model. In this context, the swelling capability of hydrogels plays a vital role in facilitating nutrient and waste diffusion, which enhances cell communication, promotes better cell growth, and strengthens the barrier function supporting essential cellular activities [9,13,57].

Various factors influence hydrogels’ swelling ability, including surface properties and incorporated signaling molecules. Hydrogels that are hydrophilic, possess large pores, and have a high density generally exhibit a higher swelling rate. However, excessive swelling can disrupt the spatial arrangement of the printed material, potentially compromising the integrity of the engineered tissue [58,59,60].

**Figure 5:**
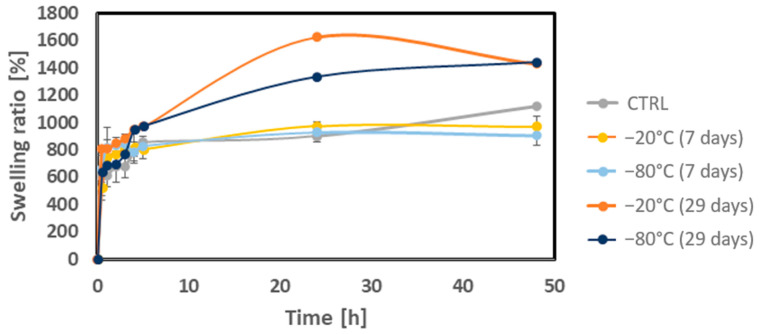
Swelling test measurements of the AMN-based hydrogel. The graph shows the measurements for the CTRL hydrogel, which is the fresh hydrogel, and the hydrogels frozen for 7 or 29 days at −20 °C and −80 °C.

Figure 5 shows that all the scaffolds, regardless of the freezing duration, exhibit an upward trend in their swelling rate over time. The selected polymers, ALG, MC, and NFC, used to form the hydrogels contain numerous polar groups, such as hydroxyl and carboxyl groups, which can form hydrogen bonds with water. This characteristic enhances the hydrogels’ capacity to absorb water [59].

The CTRL hydrogel swelled to 1115% of its initial weight and did not reach equilibrium within 48 h. In contrast, the hydrogels frozen for 7 days at −20 °C and −80 °C exhibited less swelling, reaching over 900% of their initial weight. These samples appeared to reach an equilibrium swelling ratio and began to degrade slowly after 24 h, likely due to increased scaffold permeability, which indicates degradation.

The hydrogels frozen for 29 days at −20 °C and −80 °C followed a similar swelling profile, but their mean swelling rates were significantly increased at all the intermediate time intervals. The hydrogel frozen for 29 days at −20 °C swelled to 1624% of its initial weight, while the hydrogel frozen for 29 days at −80 °C swelled to 1333% of its initial weight. Both samples reached equilibrium after 24 h.

The increasing swelling rate observed with longer freezing times may be attributed to greater water absorption, which enhanced the formation of hydrogen bonds and increased the cross-linking density within the hydrogel [61].

### 3.4. In Vitro Degradation of the 3D-Bioprinted Scaffolds

The cross-linking density largely influences the degradation rate and mechanical stiffness of hydrogels. A higher cross-linking density results in greater stiffness and slower degradation, whereas a lower cross-linking density leads to faster absorption and more rapid dissolution in aqueous environments. For biopolymers used in in vitro skin models, it is crucial to balance the degree of cross-linking. The biopolymer should not dissolve too quickly, but it should biodegrade at a pace that minimizes the environmental impact. Achieving this balance allows the hydrogel to absorb liquids and maintain its structural stability while undergoing moderate degradation. For in vitro models, the degradation profile of the hydrogel scaffold should align with the rate of cell proliferation [13,62,63,64].

All the AMN scaffolds were cross-linked after printing to retain shape fidelity and ensure an appropriate degradation rate using a 1% CaCl_2_ solution [65]. The degradation kinetics were studied to evaluate the structural integrity of the scaffolds. The degradation rate of all the tested scaffolds was monitored by measuring their weight changes over a predefined period, while incubated in ADMEM supplemented with 5% FBS at 37 °C. This approach was chosen because the scaffolds were intended for future cell inclusion and would be exposed to the cell medium for an extended period.

**Figure 6:**
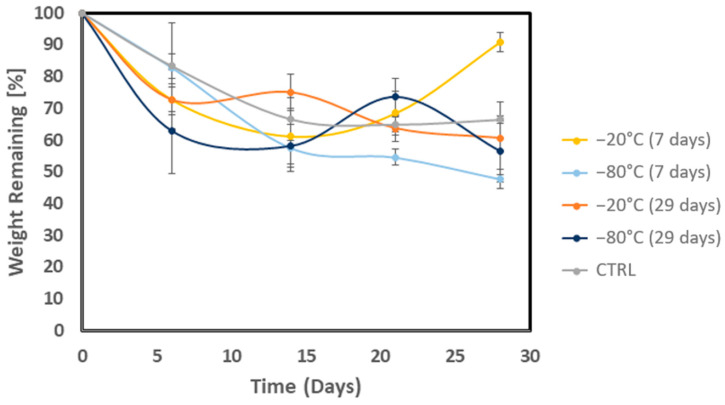
Measurements of the in vitro degradation rate of the AMN-based hydrogel. The graph shows the measurements for the CTRL hydrogel, which is the fresh hydrogel, and the hydrogels frozen for 7 or 29 days at −20 °C and −80 °C.

The results are summarized in Figure 6. The weight of all the scaffolds drops rapidly during the first few days of incubation. This initial decrease is likely due to cation exchange, where calcium ions from the scaffold are exchanged for sodium ions from the growth medium, leading to the dissolution of ALG [5,13,66]. Another possible explanation for the initial weight loss could be the loss of some of the hydrogen bonds between the pre-frozen AMN hydrogel and a decline in the cross-linking density, impairing its mechanical stability [67]. As shown in the measured swelling ratio (Figure 5), the swelling ratio reaches equilibrium after 24 h. It can be assumed that the AMN-based hydrogel gradually deforms due to the internal stress and impairment of the AMN-based hydrogel network after this point.

After 10–15 days of incubation, the weight of the scaffolds stabilizes, retaining approximately 60–75% of their initial mass for the remainder of the period. Interestingly, some of the scaffolds even show a slight increase in weight. This phenomenon could be due to the expansion of the free spaces within the scaffold structure, caused by the gradual loss of hydrogen bonds, cross-linking among the ALG molecules, and the internal stress within the scaffolds. The growth medium may occupy these enlarged spaces (ADMEM + 5% FBS). However, as the scaffolds were incubated in a limited ADMEM + 5% FBS volume, their weight eventually decreases once the medium is depleted [5,68].

In conclusion, the weight of all the scaffolds, regardless of freezing time, decreased over time, like the CTRL hydrogels. The freezing time of the scaffolds influenced both their long-term stability and degradation kinetics at every intermediate time point. From the graphs it is clear that, consistent with the observations of the swelling behavior, the hydrogels frozen for 29 days at −20 °C and −80 °C were the most negatively affected. This effect can likely be attributed to the internal stress induced by the prolonged freezing of the ALG chains in the hydrogels, which led to an altered rate and extent of the diffusion of calcium ions into the polymer matrix. A reduced cross-linking ratio usually correlates with increased porosity, weakening the hydrogel [5,68,69]. This, in turn, contributed to the degradation rate of the pre-frozen samples.

### 3.5. Characterization of 3D-Printed Scaffolds

**Figure 7:**
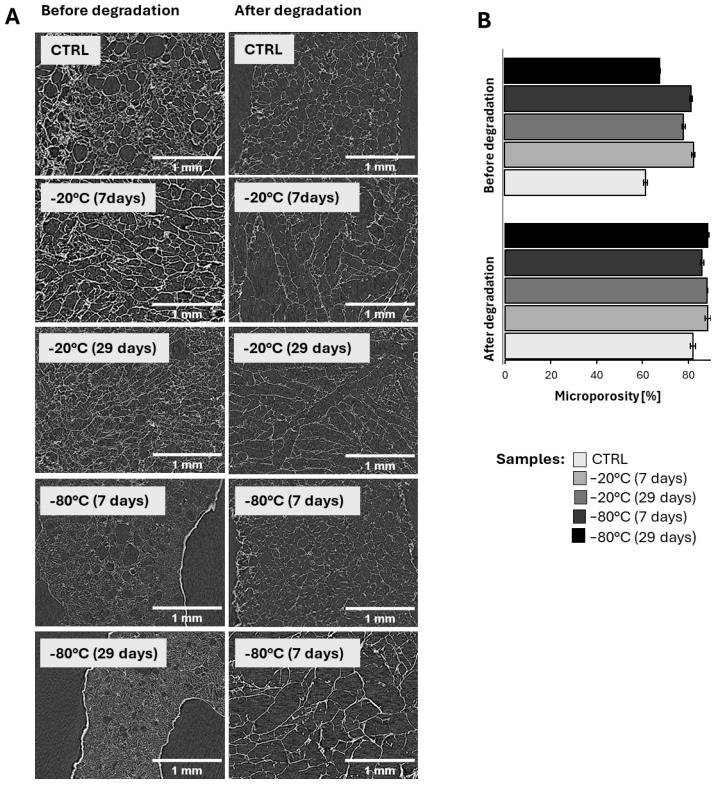
(**A**) nanoCT images of 3D-printed scaffolds, before and after degradation, printed using fresh (control) and pre-frozen hydrogels stored at different temperatures. (**B**) calculated microporosity from nanoCT images before and after degradation using ImageJ software, with each shade of grey representing a different freezing temperature and time.

The nanoCT analysis provided detailed insights into the materials’ surface characteristics and internal microstructure, focusing on the materials’ porosity (Figure 7). The images were analyzed using ImageJ (1.54g) software to determine how storing the materials at different temperatures before printing affected the porosity of the materials before and after degradation. The results of the porosity analysis are presented in Figure 7.

The nanoCT images reveal a statistically significant increase in macro- and microporosity after degradation. As shown in Figure 7, the most pronounced increase in microporosity was observed in the CTRL sample (from 60.9 ± 0.8% before degradation to 82.0 ± 1.0% after degradation) and the −80 °C (29 days) sample (from 66.8 ± 0.3% before degradation to 88.4 ± 0.6% after degradation). In contrast, the smallest increase in microporosity occurred in the −80 °C (7 days) sample (from 80.4 ± 0.5% before degradation to 86.0 ± 0.7% after degradation).

When comparing the scaffolds printed from the pre-frozen hydrogels to those printed from the fresh hydrogels, a statistically significant difference in porosity was found both before and after degradation. The higher initial microporosity observed in the pre-frozen samples suggests that ice crystal formation during freezing damaged the hydrogel network, making it mechanically fragile [17,70]. The consistent internal porosity of the −80 °C samples correlates with their degradation rates. Generally, the degradation rate is inversely proportional to a material’s cross-link density. The lower cross-link density of the −80 °C samples likely contributed to their higher initial microporosity and the observed lower degradation rate (Figure 6). This phenomenon may have implications for the structural and functional properties of the 3D-bioprinted skin model. The surface characteristics and internal microstructure were further analyzed using SEM.

**Figure 8:**
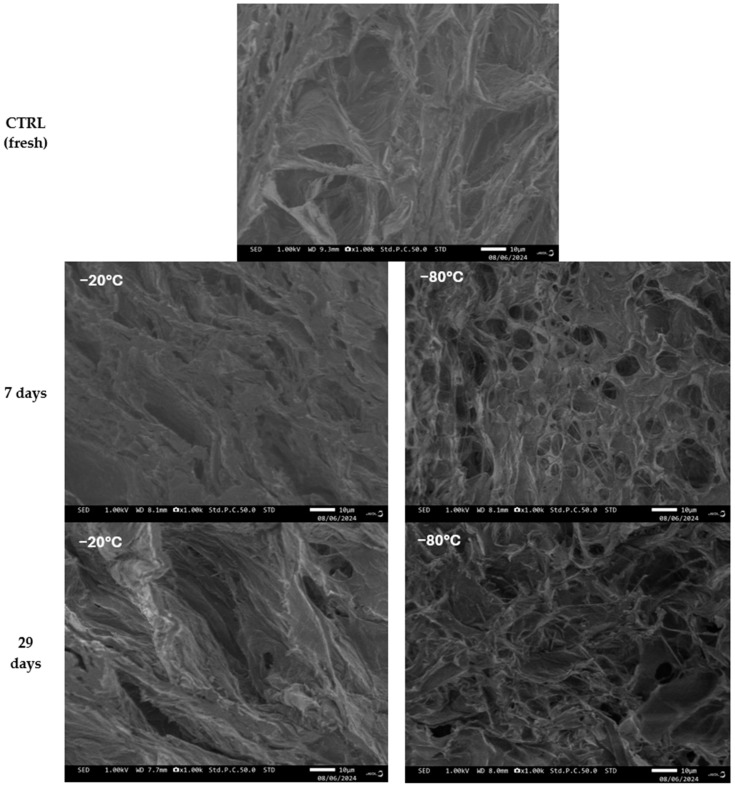
Cross-section SEM images of 3D-printed scaffolds comparing fresh CTRL hydrogel and frozen hydrogels stored at different temperatures (−20 °C and −80 °C) and durations (7 days and 29 days).

The SEM analysis (Figure 8) corroborates the findings from the nanoCT and highlights the significant alterations in both the micro- and macroporosity of the scaffolds under the investigated conditions. The micrographs reveal an increase in scaffold porosity in the pre-frozen hydrogels, with this effect being particularly pronounced in the samples frozen at −80 °C. The observed macroporous gel morphology is influenced by phase separation between the frozen solvent (i.e., the ice formed from the water content in the hydrogels) and the reactive gel precursor solution (i.e., the cross-linker solution). The freezing rate and temperature are crucial for ice formation and growth, impacting gel porosity. Specifically, lower temperatures and faster ice nucleation kinetics result in gels with smaller pore sizes [17,70].

Significant morphological changes were also noted on the pore walls of the hydrogels stored at −80 °C. These pore walls appear less compact, potentially increasing their accessibility for the diffusion of low-molecular-weight bivalent cations [17,70]. This suggests that the freezing temperature strongly influences the morphology of the hydrogel network’s pore walls. Additionally, the samples frozen at −80 °C exhibited a more heterogeneous morphology, characterized by hexagonal-like structures, compared to those frozen at −20 °C and the CTRL hydrogel, which displayed a more uniform structure. The presence of these hexagonal structures suggests distortion caused by ice crystal growth [17,70].

Moreover, the freezing time significantly affects the arrangement of the polymer network, likely due to the phase transition of the hydrogel from liquid to solid and the accompanying phase separation during this process. Since the freezing rate is slower at the center of the sample than at the edges, crystallization initiates at different times within the sample [17,70]. Prolonged freezing leads to further ice crystal formation and growth in the core, resulting in a more porous structure after thawing [17,70]. The increased porosity of the pre-frozen samples has implications for their swelling capacity and degradation kinetics, which are discussed in more detail in Section 3.2. and Section 3.3.

### 3.6. Mechanical Properties of 3D-Printed Scaffolds

Hydrated materials, like hydrogels, are composed of water embedded within a 3D network of covalently cross-linked polymer chains. This structure results in mechanical behavior that is significantly influenced by the hydrogel scaffold’s intrinsic elastic properties and the water’s time-dependent movement through the deformed structure, a phenomenon known as poroelasticity. This interaction can significantly influence cell growth and morphology [71].

The results of the nanoindentation tests, presented in Table 1, exhibit a large standard deviation. This variability is likely due to the small area of the individual indents, the potential inhomogeneity of the pre-frozen samples, and the challenges encountered during the 3D bioprinting process. These factors may contribute to the inconsistency observed across the different test points.

Interestingly, the results indicate that freezing at −20 °C, especially for the samples frozen for 7 days, led to an unexpected improvement in the hardness and elasticity of the hydrogel. This enhancement is likely due to a reduction in porosity, which strengthens the polymer chain bonds, thereby increasing the stiffness and hardness of the material [72]. In addition, the similar values observed for the plastic and elastic work suggest that freezing at −20 °C may have a less time-dependent impact on the poroelasticity than freezing at −80 °C [73]. The smaller effect on the poroelasticity at this temperature could be attributed to a more uniform pore size and distribution, reducing the water movement’s influence on the mechanical properties.

In contrast, the reduction in hardness and elasticity observed in the samples pre-frozen at −80 °C can be explained by the greater spacing between the polymer chains and increased internal porosity, which are consequences of ice crystal formation during the freezing process, as evidenced in Figure 7 and Figure 8. The formation of larger ice crystals at this lower temperature could lead to more significant disruptions in the hydrogel network, resulting in weaker mechanical properties.

Contrary to expectations, the CTRL sample, hypothesized to exhibit the best mechanical properties due to reduced porosity, displayed deviations in the results. This anomaly could be attributed to the inhomogeneous distribution of the NFC fibrils within the sample and their potential agglomeration [72,73]. Moreover, using low forces and small surface areas for the Berkovich indenter tests may have introduced high variability, as the indenter could have come into contact with individual filaments, fiber agglomerates, or pores, leading to hardness values that varied widely, sometimes approaching zero [72,73].

## 4. Conclusions

In this study, we investigated the impact of freezing on the physicochemical properties of a hydrogel used for 3D-bioprinted skin models to enable consistent and reliable testing during the development of an in vitro skin model. By preparing a large batch of hydrogel and storing it by freezing, we aimed to use the exact same formulation across multiple tests, thereby eliminating the variations that can occur with each new preparation.

Our findings revealed that freezing significantly affected the hydrogel’s properties, particularly its viscosity and porosity, which influenced the extrusion process and the structural integrity of the 3D-bioprinted scaffold. Notably, significant changes were observed in the samples stored at −80 °C, where prolonged freezing times led to increased ice crystal formation, decreased viscosity, and increased porosity. These changes compromised the scaffold’s morphology, water absorption, and degradation kinetics, potentially affecting the 3D-bioprinted skin model’s ability to mimic native skin conditions. In contrast, the samples stored at −20 °C were much less affected by the freezing process.

Despite these challenges, our study suggests that with the careful optimization of the freezing temperature and storage duration, it may be possible to identify the conditions that would allow for a single batch of hydrogel to be used consistently across all the tests required during in vitro skin model development without significantly altering its properties. This would ensure consistency and reliability throughout the development process. However, additional testing is necessary to confirm these findings and to fine-tune the storage conditions to achieve the desired consistency and reliability of the hydrogel’s performance.

## Figures and Tables

**Table 1:** Nanoindentation mechanical properties of the tested 3D-bioprinted scaffolds.

Nanoindentation Mechanical Properties				
	Hardness [MPa]	Indentation Modulus of Elasticity [MPa]	Plastic Work [nJ]	Elastic Work [nJ]
CTRL	26 ± 19	585 ± 239	54.4 ± 19.6	27.1 ± 5.3
−20 °C (7 days)	229 ± 109	2971 ± 983	12.8 ± 3.6	10.6 ± 1.7
−20 °C (29 days)	59 ± 14	1062 ± 230	22.3 ± 4.4	20.8 ± 3.5
−80 °C (7 days)	20 ± 7	249 ± 45	43 ± 11	41.5 ± 3.3
−80 °C (29 days)	28 ± 17	644 ± 240	49.3 ± 18	28.1 ± 6.4

## Data Availability

The original contributions presented in the study are included in the article, further inquiries can be directed to the corresponding author.
